# The production of UL16-binding protein 1 targeted pigs using CRISPR technology

**DOI:** 10.1007/s13205-018-1107-4

**Published:** 2018-01-13

**Authors:** Zeyland Joanna, Hryhorowicz Magdalena, Nowak-Terpiłowska Agnieszka, Jura Jacek, Słomski Ryszard, Smorąg Zdzisław, Gajda Barbara, Lipiński Daniel

**Affiliations:** 1 Department of Biochemistry and Biotechnology, Poznan University of Life Sciences, Dojazd 11, 60-632 Poznań, Poland; 2 Department of Animal Reproduction, National Research Institute of Animal Production, Krakowska 1, 32-083 Balice, Poland; 3 Institute of Human Genetics, Polish Academy of Sciences, Strzeszyńska 32, 60-479 Poznań, Poland

**Keywords:** CRISPR-Cas9, Microinjection, KO pigs, Xenotransplantation

## Abstract

Two sgRNAs were designed to target the region of exon 2 of the pULBP1 gene by microinjection. The co-injection of modified Cas9-D10A nickase with a pair of sgRNAs into the zygote’s cytoplasm easily and efficiently generated biallelic modification of the pULBP1 gene in one step. Five out of nine F0 generation piglets showed insertions or deletions in the targeting site of the pULBP1 gene, indicating that pULBP1 mutation efficiency reached about 56% (5/9). Quantitative determination of pULBP1 showed approximately a 1.53-fold reduction in the amount of protein ULBP1 on the cell surface (ELISA). A human NK-cell cytotoxicity test leads to the conclusion that higher cell viability is observed for −/− ULBP1 (survival rate 85.36%) compared to +/+ ULBP1 (69.58%). ULBP1-KO pigs will provide a more progressive xenograft source for further research studies, especially those measuring the effects of abolishing the gene function in terms of the complexity of the immunological interactions.

## Introduction

A donor shortage is the main limitation for conducting transplantations to save patients or improve their quality of life by treating severe organ failure. Despite improvements in the organ registration and distribution systems, in reduced toxicity of immunosuppressive treatments and even in education by means of social campaigns increasing the number of cadaveric and living donors, the gap between the number of organs available and demand must be filled. The challenges in cross-species transplantation have been considered for at least 300 years (Cooper 2012). Non-human primates (NHPs) representing phylogenetically concordant species to humans are consequently being excluded, because their medical utility provides both strong ethical resistance and transmission of xenozoonoze threats. Numerous attributes support the use of discordant pigs to meet the shortfall in organs. At the same time, the large phylogenetic distance casts a shadow over pig-to-human xenotransplantation. The organs from non-genetically engineered pigs (wild-type) are useless for medical applications because of massed antibody- and complement-mediated rejection (Cooper et al. 2016). The cells of grafted organs encounter xenorejection triggered by different types of immunological response. Hyperacute rejection (HAR) resulting in a procoagulant phenotype of swine endothelium leads to thrombosis and immediate (within minutes) loss of organ function (Pierson et al. 2009). The main cause of hyperacute rejection is an antigen present in the glycolipids and glycoproteins of the endothelial cells surface of pigs (not present in humans nor in NHPs). Carbohydrate residue Galα1,3Galβ-R (αGal) synthesis is catalysed by α1,3-galactosyltransferase (α1,3GT, EC2.4.1.151) and results from adding galactose residue to N-acetyllactosamine. Another significant problem is the incompatibility of porcine complement regulatory proteins (CRPs) on donor endothelium with the human complement, which results in uncontrolled complement activation. HAR silencing by immunosuppressive therapies and the organs from genetically modified pigs with α1,3-galactosyltransferase knockout (GTKO) or/and expressing human α1,2-fucosyltransferase (hHT), or/and galactosidase (GLA), or/and expressing human complement regulatory proteins (hCRPs) will also reveal cellular and/or humoral epiphenomena causing delayed responses, including xenograft rejection (DXR), also called acute humoral xenograft rejection (AHXR) (Cooper 2012, Puga Yung et al. 2017). Acute vascular rejection (AVR) is induced by xenoreactive antibodies directed against the endothelium of blood vessels and partially by complement activation. Endothelium–xenoantibody interactions induce (1) the production of interleukin IL-1α, which stimulates tissue factor, plasminogen-activator inhibitor type 1 (PAI-1), E-selectin and thromboxane A_2_ (TXA_2_) secretion, and (2) small amounts of complement or platelets, leading to vasoconstriction, thrombosis, and inflammation, which are characteristic of AVR. At the same time, endothelial cells are lost by apoptosis, limiting the availability of nitric oxide (NO) (Cascalho and Platt 2001). Small genetic differences found between the recipient and the donor of the transplanted organ may also lead to a disturbance in the equilibrium in proanticoagulant–anticoagulant activity of the coagulation system, which subsequently triggers xenograft rejection (Boksa et al. 2015). Coagulation dysregulation can result in thrombotic microangiopathy, causing thrombus occlusion of organ vasculature, leading to ischemic necrosis (Cooper et al. 2015). Vascularized grafts are lost because of AHXR characterized by transcriptional endothelial cell activation, thrombosis with fibrin deposition, and infiltration by innate immune cells (natural killer cells, monocyte/macrophages, and neutrophils) (Rieben and Seebach 2005, Cooper et al. 2015). Pig organs ex vivo perfused with human blood are infiltrated by NK cells (Lilienfeld et al. 2006). Activated NK cells are capable of destroying the xenografts by direct cytotoxic effect regulated by the predominance of positive (activating lytic mechanisms) or negative (inhibiting reaction) signals or antibody-dependent cellular cytotoxicity (ADCC). UL16-binding protein 1 (pULBP1) on porcine endothelium cells was shown to be the main functional ligand for the C-type lectin human NKG2D receptor on NK cells (Lilienfeld et al. 2006). The elimination of potential porcine ligands for receptors activating NK cells and, on the other hand, increasing the number of ligands for inhibitory receptors might prevent xenograft rejection involving NK cells. According to Pierson et al., strategies to overcome NK-cell activity also include human MHC class I molecules expression on the surface of swine endothelium and blocking of molecular events leading to NK recruitment (Pierson et al. 2009).

Fourteen years ago, when the first piglets with the biallelic KO phenotype of GGTA1 were born at PPL Therapeutics, molecular techniques were limited. Since then, new technologies involving zinc finger nucleases (ZFNs), transcription activator-like effector nucleases (TALENs), and RNA-guided endonucleases (CRISPR) have evolved. CRISPR-Cas9 (clustered regularly interspaced short palindromic repeats/CRISPR-associated) adaptive immune systems constitute a bacterial defense against invading nucleic acids derived from bacteriophages/plasmids. Single-guide sgRNAs hybridize and form a complex with Cas9 nuclease, which recognizes and cleaves genetic material. Imperfect repair of double-strand breaks (DSBs) results in the modification of the targeted site. Techniques based on CRISPR-Cas9 are simple and quick tools for genome editing, also adapted to create modified pigs for xenotransplantation. In this study, we present a generation of pigs with pULBP1 gene modified by CRISPR-Cas9 and their potential use as donors in xenotransplantation.

## Methods

### Cas9-D10A/gRNA design

Two sgRNAs were designed to target the region of exon 2 of the pULBP1 gene. The first sgRNA binds to GGTGTCCAGGGCTGGGCTTTGG and the second to the TGGTGTGTGGTTCAAGCCCAGG target site. The sgRNAs in the RNA format of the final concentration of 200 ng/µl were provided and validated by Sigma-Aldrich. The Cas9-D10A was also provided by Sigma-Aldrich in the mRNA format with a final concentration of 500 ng/µl.

### Microinjection

Superovulation was achieved by use of standard protocol (Jura et al. 2007). Zygotes were recovered by flushing the oviducts from donor gilts under full anesthesia. After collection, zygotes were morphologically evaluated. Only zygotes with intact cytoplasm and two visible polar bodies were subjected to transfection performed by intracytoplasmic microinjection of the gRNAs and mRNA of Cas9-D10A. Microinjection was performed under an inverted microscope equipped with Nomarski’s optics assisted by two micromanipulator units. The Cas9-D10A mRNA working concentration was 35 ng/μl. The concentration of each gRNA was 30 ng/µl. After microinjection, the microinjected zygotes were transferred to a chamber containing an albumin-supplemented solution and evaluated morphologically again to eliminate any damaged zygotes. Positively evaluated zygotes were transferred into the fallopian tubes of synchronized recipient gilts checked for signs of oestrus. Transfer procedures were performed under full surgical anesthesia. The oviducts were pulled out through a 2–3 cm-long incision made along the white lines. Microinjected zygotes suspended in PBS supplemented with 20% FCS were expelled into the oviduct by the insertion of a fine plastic cannula connected to a Hamilton syringe. Recipient gilts were checked for pregnancy after 30 days.

### CAS9/gRNA mutation screening and sequencing

Genomic DNA was extracted from ear biopsy specimens of pigs using a Kapa Express Extract DNA Extraction Kit with a PCR ReadyMix (*KAPA BIOSYSTEMS*). PCR was performed using ULBP1-F (5′-CTC ACC TGC GTT TTG CCT TC-3′) and ULBP1-R (5′-CCT TGA GGA AGT CCC CAA CG-3′) primers under the following conditions: 94 °C for 180 s, followed by 35 cycles of 94 °C for 30 s, 58 °C for 45 s, 72 °C for 30 s, and a final extension at 72 °C for 360 s. The 246-bp PCR product was directly sequenced using an automated genetic analyzer (Applied Biosystems Prism) with a ULBP1-R primer. The 246-bp PCR products from different individuals obtained with ULBP1-F and ULBP1-R primers that showed in direct sequencing mixed base calls (overlapped peaks in the sequencing chromatographs) were cloned into a pSC-A-amp/kan cloning vector (Agilent Technologies), according to the manufacturer’s protocol. The white colonies were selected for recombinant plasmid analysis. Plasmid DNA minipreps were prepared according to the protocol of the Stratagene Miniprep Kit (Agilent Technologies) and sequenced using an automated genetic analyzer (Applied Biosystems Prism) using an M13F primer (5′-CGCCAGGGTTTTCCCAGTCACGAC-3′).

### NK-cell culture

An interleukin-2 independent human natural killer cell line (NK92MI—*ATCC*) was cultured in Alpha Minimum Essential Medium supplemented with 2 mM l-glutamine, 1.5 g/L sodium bicarbonate, 0.2 mM inositol, 0.1 mM 2-mercaptoethanol, 0.02 mM folic acid, horse serum to a final concentration of 12.5%, fetal bovine serum to a final concentration of 12.5, and 1% antibiotic/antimycotic. Cultures were maintained by centrifuging cells and resuspending cell pellets in a fresh medium at 2–3 × 10^5^ viable cells/ml.

### Porcine aortic endothelial cells (PAEC)

Primary porcine aortic endothelial cells were obtained in our laboratory and were isolated from modified and +/+ ULBP1 pigs. To isolate endothelial cells (8–10 cm), fragments of aorta (8–10 cm) were washed three times in PBS (Phosphate Buffered Saline). The piece was then placed on a sterile tray. The ends of the aorta were spun with umbilical cord clips. Aorta was filled with collagenase IV through the artery intercostal hole and placed in an incubator at 37 °C for 10 min. After incubation, the cell suspension inside the aorta was transferred to a 15 ml centrifuge tube. Any remaining endothelial cells in the aorta were scraped with a sterile swab and rinsed with collagenase. 5 ml of the previously prepared culture medium for endothelial cells was added (RPMI 1640, 10% FBS (Fetal Bovine Serum), 1% antibiotic, endothelial cell growth factor, heparin) to the cell suspension and centrifuged at 1200 rpm for 5 min at room temperature. After centrifuging, the supernatant was removed and the cells were resuspended in 3 ml of the culture medium. The cells were transferred to 6-well culture plates coated with 0.1% gelatin. The culture plates were placed in an incubator at 37 °C, 5% CO_2_ atmosphere. After 24 h, the medium was changed. After 72 h, the cells were moved and grown in 25 cm^2^ culture vessels. The cells were cultured until they reached 80–90% confluency. After reaching the proper confluence, passage was performed.

### Cytotoxicity assays

To evaluate the effect of disruption to the pULBP1 gene, a human NK-cell cytotoxicity test was performed. NK-92MI cells were cocultured with endothelial cells derived from −/− ULBP1 pig #184 and with +/+ ULBP1 endothelial cells as the control. 100 µl of an endothelial cell suspension (5000 cells/well) was dispensed in a 96-well plate as target cells. After 24 h pre-incubation in complete medium (RPMI 1640, 10% FBS, 1% antibiotic, endothelial cell growth factor, heparin) in a humidified incubator (37 °C, 5% CO_2_), the medium was removed and human NK cells were added to the target cells. The 5:1 effector cell-to-target cell (E:T) ratios were used in each experiment. These cells were cocultured for 4 h in a complete growth medium for NK cells (MEM with 2 mM l-glutamine, 1.5 g/L sodium bicarbonate, 0.2 mM inositol, 0.1 mM 2-mercaptoethanol, 0.02 mM folic acid, 12.5% horse serum, and 12.5% fetal bovine serum). The cytotoxic activity of the human NK-cell lines NK92MI was tested in sensitive colorimetric assays using a Cell Counting Kit-8 (Sigma-Aldrich). The Cell Counting Kit-8 (CCK-8) allows assaying with the use of water soluble tetrazolium salt (WST-8). WST-8 is reduced by mitochondrial dehydrogenases to an orange formazan, which is soluble in the culture medium. The amount of formazan dye is directly proportional to the number of viable cells. CCK-8 allows sensitive colorimetric assays for the evaluation of the number of living cells in the cytotoxicity assays. After 4 h incubation, 10 µl of the CCK-8 reagent was added to each well, and the cells were incubated for 2 h at 37 °C. The spectrophotometric absorbance of each sample was measured using a microplate reader at 450 nm. The percentage of surviving cells in each group tested was calculated. These experiments were carried out two times with four replicates for each animal. Control cell lines were derived from two different +/+ ULBP1 individuals.

### ELISA assay

Quantitative determination of pULBP1 was conducted with a ULBP1 ELISA kit (BlueGene, catalogue number: E07U0022). This kit uses the competitive enzyme immunoassay technique utilizing a monoclonal anti-ULBP1 antibody and a ULBP1-HRP (horseradish peroxidase) conjugate. The assay sample (cell culture supernatants) and buffer were incubated together with the ULBP1-HRP conjugate on a pre-coated plate for 1 h. After the incubation period, the wells were decanted and washed five times. The wells were then incubated with a substrate for HRP enzyme. The product of the enzyme–substrate reaction formed a blue-colored complex. Finally, a stop solution was added to stop the reaction, which turned the solution yellow. The intensity of color was measured spectrophotometrically at 450 nm in a microplate reader. The intensity of the color was inversely proportional to the ULBP1 concentration, since ULBP1 from samples and ULBP1-HRP conjugate competed for the anti-ULBP1 antibody binding site. Because the number of sites was limited, as more were occupied by ULBP1 from the sample, fewer sites were left to bind the ULBP1-HRP conjugate. A standard curve was plotted to show the intensity of the color (O.D.) to the concentration of standards. The ULBP1 concentration in each sample was interpolated from this standard curve.

## Results

**Fig. 1:**
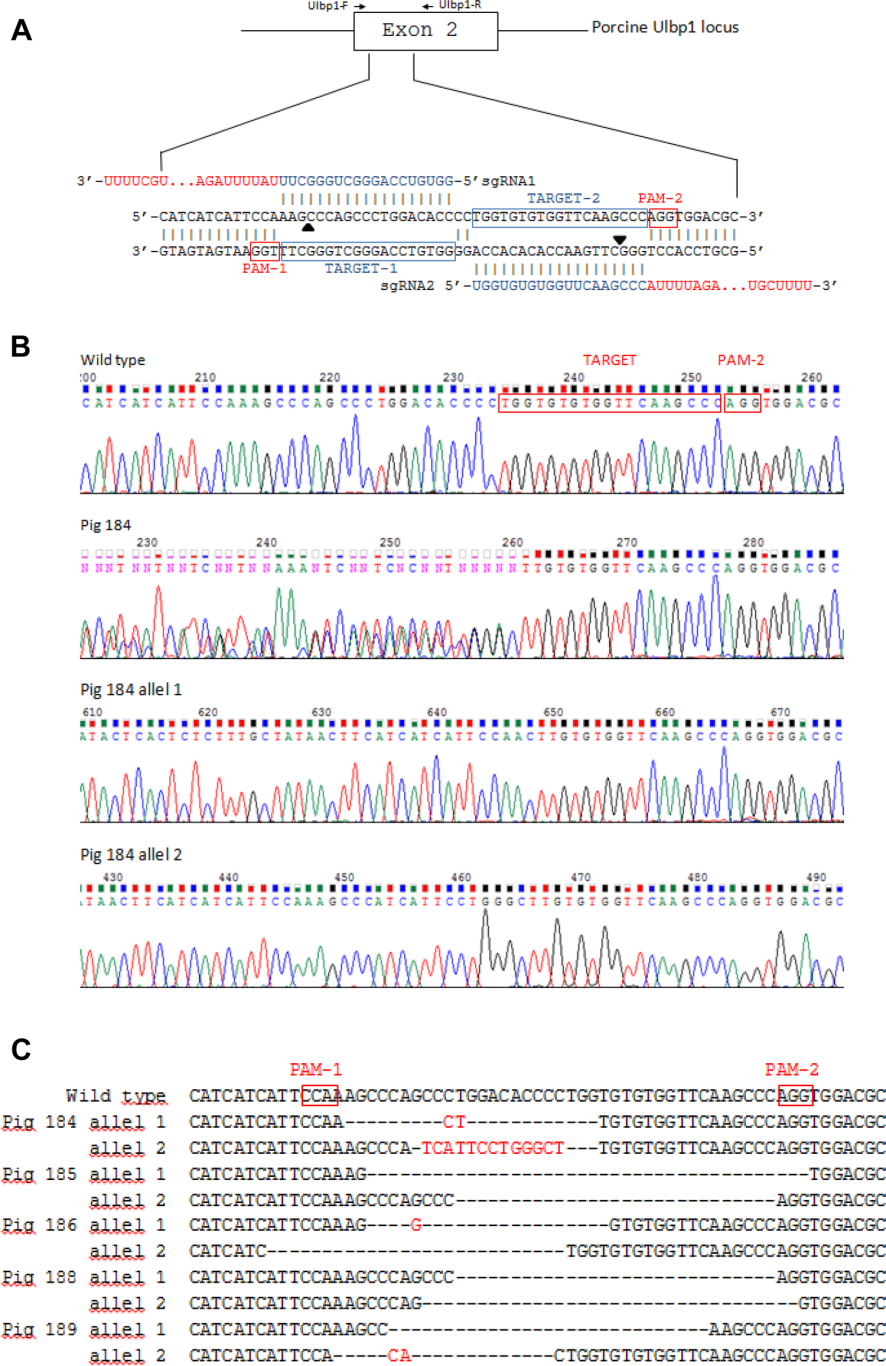
Generation of UL16-binding protein 1 targeted pigs using CRISPR-Cas9 technology. **a** Schematic representation of nucleotide sequences between the target locus (exon 2 of ULBP1) and ULBP1—targeting sgRNAs. The black arrows indicate the putative cleavage site. The primers used are shown above the sequence of ULBP1. **b** Direct sequencing of PCR products from +/+ ULBP1, −/− ULBP1 pig #184 and alleles separated by cloning. **c** List of mutations. The inserted base is marked in red. Black bars indicate deletion of bases. PAM-1 and PAM-2, protospacer adjacent motifs

Two sgRNAs were designed to target the region of exon 2 of the UL16-Binding Protein 1 (pULBP1) gene (Fig. 1a). A mix of Cas9-D10A mRNA and a pair of sgRNAs was introduced into cytoplasm of the zygotes based on the microinjection procedure applied previously (Jura et al. 2007). Seventy-three fertilized egg cells from three donors were subjected to intracytoplasmic microinjection. 100% of the microinjected zygotes were qualified for transfer after a morphological examination, which suggests that sgRNAs/Cas9-D10A and the microinjection manipulation had no negative influence on the early embryonic development.

The injected embryos were transferred into the oviducts of two synchronized surrogates (36/37 each). Both surrogates were pregnant and delivered a total of nine piglets (F0 generation). Piglets were subjected to Cas9/gRNA mutation screening to detect CRISPR-induced mutations. The 246-bp PCR products obtained from different individuals that showed in direct PCR sequencing mixed base calls (overlapped peaks in the sequencing chromatographs) were cloned (Fig. 1b). Plasmid DNA was sequenced. The analysis showed four F0 generation piglets with +/+ ULBP1genotype (4/9, 44%). Five females showed insertions or deletions (indels) in the targeting site of exon 2 of the pULBP1 gene, indicating that pULBP1 mutation efficiency reached about 56% (5/9). All pigs were altered in a biallelic manner, showing different sequences in each mutant allele (5/5). We did not observe more than two different alleles in each individual, which, to some extent, contradicts the results obtained by other researchers (Geurts et al. 2009; Hai et al. 2014). Two piglets (#185 and #188) had both alleles with frameshift. Hence, both are homozygous. Two piglets (#184 and #189) are heterozygous, because only one allele had frameshift. Piglet #186 had none allele with frameshift.

Our studies suggest that Cas9-D10A nicking occurred during the one-cell stage of embryo development. Two piglets (pig#185 and #188) represented the same sequence of one mutant allele (Fig. 1c).

**Fig. 2:**
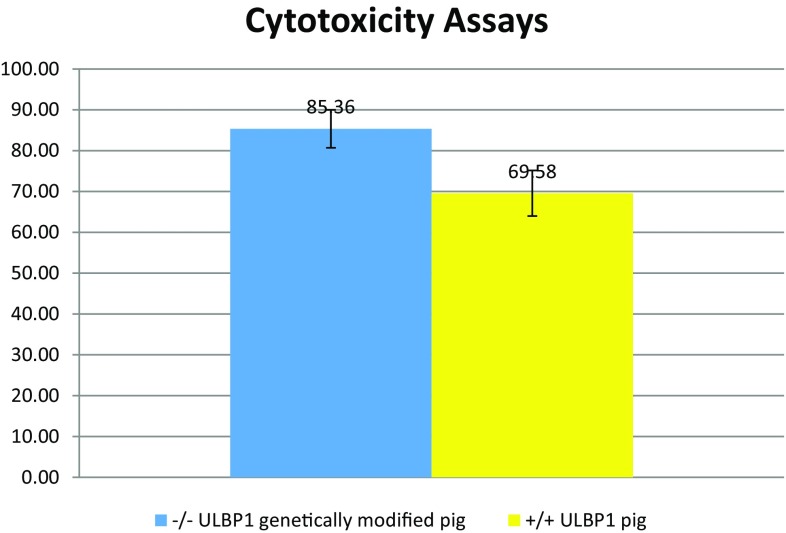
Survival rate analysis for cells from −/− ULBP1 pig #184 (blue) and +/+ ULBP1 pig (yellow) in the medium with human NK cells. The human NK cells (effector cells) were added to endothelial cells (target cells) in an E:T ratio 5:1. After co-culture for 4 h, CCK-8 was used to detect the remaining vital cells. The survival rate was measured by the average number of living cells. The percentage of surviving cells in each tested group was marked on Y-axis. Means were compared by Welch two-sample *t* test (*p* value = 0.0565)

The functional effect of pULBP1 gene disruption in the cell lines was tested in NK-cell cytotoxicity assays using +/+ ULBP1 pig endothelial cells as a control. To test whether −/− ULBP1 pig #184 endothelial cells were protected from xenogeneic human NK cytotoxicity, we performed assays using the endothelial cells as targets and human NK-cell lines NK92MI as effector cells. The survival rate was determined by CCK-8 colorimetric assays for the determination of cell viability in cell cytotoxicity assays. The results of measuring the absorbance of cells after incubation with human NK cells lead to the conclusion that higher cell viability is observed for -/-ULBP1 (survival rate 85.36%) compared to +/+ ULBP1 (69.58%) (Fig. 2).

**Fig. 3:**
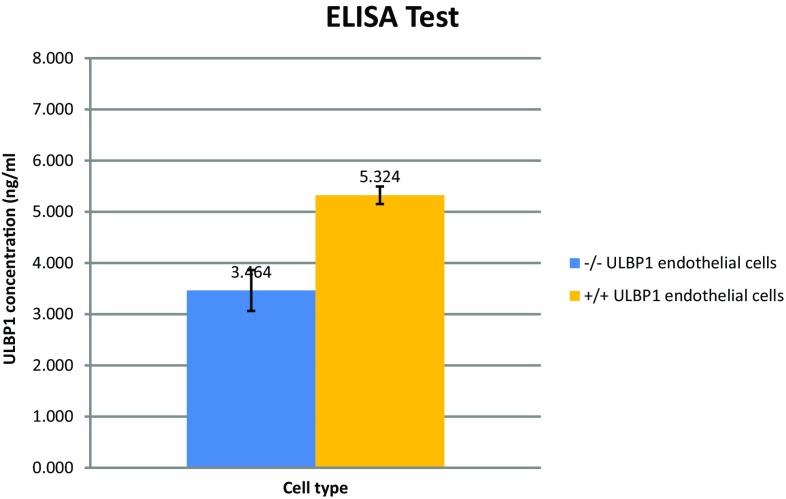
ULBP1 concentration level in the culture medium was detected using an ELISA assay—the competitive enzyme immunoassay technique utilizing a monoclonal anti-ULBP1 antibody and an ULBP1-HRP conjugate. Analysis was conducted for cells from −/− ULBP1 genetically modified pig #184 and +/+ ULBP1 pig. The ULBP1 concentration was marked on the Y-axis. Presented results are from one of three independent experiments. Means were compared by Welch two-sample *t* test (*p* value = 0.00376)

In an effort to confirm a reduction in pULBP1 surface expression on PAEC, ELISA assaying using monoclonal antibodies was performed. The ELISA was conducted on cell-derived supernatants: −/− ULBP1 pig #184 aortic endothelial cells and +/+ ULBP1 pig aortic endothelial cells as control. Data from spectrophotometric cell analysis were inversely proportional to the pULBP1 concentration. The pULBP1 concentration in each sample was interpolated from the standard curve. The results of absorbance measurement cells after incubation with anti-ULBP1 antibody showed approximately a 1.53-fold reduction in the amount of protein ULBP1 on the cell surface (−/− ULBP1 EC, ULBP1 concentration = 3.464 ng/ml; +/+ ULBP1 EC, ULBP1 concentration = 5.324 ng/ml). All experiments were performed three times with cells obtained from three different portions of aorta. Control cell lines were derived from three different +/+ ULBP1 individuals. The average values of the three repetitions are shown in Fig. 3.

## Discussion

In this study, we present genetically modified pigs with a disrupted UL16-Binding Protein 1 gene with CRISPR-Cas9 technology. We also describe the modification of the pULBP1 gene using Cas9-D10A nickase for the first time, showing that a pair of sgRNAs targeting opposite strands of a target locus facilitates high-efficiency pig genome modification. In this study, we fused the technology based on CRISPR-Cas9-D10A with the microinjection into the cytoplasm.

The sgRNA is able to direct the Cas9 nuclease to any genomic target site. Cas9 nuclease specificity is high, but not precise, which reduces the possibilities of editing applications. The multiple mismatches between the sequence of sgRNA and targeted DNA are accepted, leading to off-target double-strand breaks (DSBs) and indel formation. The specificity improvement of Cas9 activity was achieved by mutation of the catalytic residues of the conserved domains (D10A in RuvC) and (H840A in HNH) converting nucleases into DNA nickases (Ran et al. 2013). The opposite strand nickase double cleavages of the targeted site require the cooperation of two independent sets of complex activities of Cas9-D10A/sgRNA. This kind of action is similar to those applied in ZFNs and TALENs technologies, which suggests an increase in specificity.

DSBs are generally repaired by homologous directed repair (HDR) if the homologous template is available or otherwise by non-homologous end-joining (NHEJ). NHEJ is an error-prone process that can rapidly ligate the broken ends, but generates small insertions and deletions (indels) at targeted sites, which often results in the function of target genes being disrupted or eliminated. Alternatively, DSB may also be repaired via HDR, which can be used to introduce transgenes or precise genome editing by providing an exogenous repair template.

In our study, we applied a combination of two sgRNAs and Cas9-D10A to produce modified pigs by microinjection skipping somatic cell nuclear transfer (SCNT). SCNT is burdened with limited proliferative capacity, and the risk of producing animals with abnormalities and low yield, and hence, we decided not to apply it. Ran et al. successfully targeted the Mecp2 locus in 80% of screened embryos by a cytoplasmic co-injection of Cas9n mRNA and sgRNAs into single-cell mouse zygotes (2013). CRISPR-Cas9 technology was applied for the first time to generate pigs with von Willebrand gene knockout (vWF) but still with wild-type Cas9 and sgRNA (Hai et al. 2014). One of the first approaches to targeting the embryos of pigs was made by Wang et al. using wild-type Cas9 and one sgRNA to knock out both loci of Npc1l1 (Wang et al. 2015). To increase specificity and reduce off-targets, at the slight expense of the efficiency of the technology (56% in our study compared to 63% in Hai et al.’s study), we used modified Cas9-D10A and a pair of sgRNAs (Hai et al. 2014). Direct microinjection of mRNA of Cas9-D10A and sgRNAs should be preferable, because it shortens time, reduces costs, and does not require transfection followed by the selection of donor cells.

NK cell-mediated rejection is one of the barriers preventing the clinical application of pig-to-human xenotransplantation. Activated NK cells are able to destroy xenograft cells through direct contact between NK cells and target cells (Rieben and Seebach 2005). Direct cytotoxicity is regulated by the balance activating and inhibitory receptors of NK cells (Lopez-Botet and Bellon 1999). The signals transmitted by activating receptors lead to NK-cell activation and lysis of the target cell; then, stimulation of the inhibitory receptor leads to the inhibition of the cytotoxic reaction. The human NK cytotoxicity against porcine endothelial cells is mediated by binding porcine ULBP1 antigen (UL-16-binding protein) to the NKG2D activating receptors of NK cells. Lilienfeld et al. demonstrated that xenogeneic cytotoxicity, mediated by freshly isolated NK cells and IL-2-activated NK cells through the NKG2D receptor, was inhibited using the anti-porcine ULBP1 polyclonal antibody (2006). pULBP2 is also expressed on the porcine endothelium with very low mRNA levels, suggesting the diminished significance of this ligand. Some authors suggest that the interactions observed between hNKG2D and pULBP1 triggered by molecular compatibilities probably would be able to control interspecies pathogen transmission (Lilienfeld et al. 2006).

As expected in our experiment, the capacity of human NK cells to lyse target cells was reduced in comparison to +/+ ULBP1 cells. However, no full protection was observed. The partial abrogation of cytotoxicity could have some potential causes. In the case of our results, the level of the cytotoxicity effect on human NK cells is reduced by 16% (−/− ULBP1 pig #184 line—survival rate 85.36% compared to +/+ ULBP1 line—69.58%) and a 1.53 times decreased level of the protein ULBP1 on the cells’ surface was demonstrated by ELISA. These results confirm the moderate protective effect of disruption to the pULBP1 gene. pULBP1’s incomplete elimination from the cell surface demonstrated by ELISA is caused by the fact that only in one allele did the frameshift occur in the tested individual. The modification in the second allele did not lead to full protein dysfunction. The reason for the lower than expected reduction in the amount of ULBP1 protein on the surface of PAEC cells is probably the use in our experiment of cells from an individual who had a deletion in both gene alleles, but the change of reading frame occurred only in one of two alleles. It is very likely that in the case of the second allele, despite the deletion, a shorter protein product is produced and eventually recognized by the ELISA test. These results confirm that the amount of ULBP1 on the surface of PAEC cells is reduced. Because of the fact that the breeding process is time-consuming and expensive, animals with pULBP1 gene deletions leading to a change in reading frame in both alleles, which would be expected to fully eliminate pULBP1 protein, are in our opinion too valuable. Due to the above, they have been designed for breeding activities that enable the spread of a new trait in a larger group of animals. In addition, there is no information regarding which domain the ELISA antibody detects, because the Technical Manual only reports that the test is “designed for the quantitative determination of Porcine ULBP1” (BlueGene). Nor is there any detailed information about the mechanism of the antigen and monoclonal anti-ULBP1 antibody binding. We suppose that NK cytotoxicity is based on mechanisms associated with the other domains.

F0 generation animals are now being crossbred to produce pigs with the expected total elimination of pULBP1 from the cell surface and greater protective effect compared with those observed in this study. A further reason for the partial abrogation of the cytotoxicity effect could be the lack of the capacity to deliver inhibitory signals mediated by porcine major histocompatibility complex (MHC) class I molecules to human NK receptors. This results from molecular differences within the MHC of pigs (swine leukocyte antigens) and that of humans (human leukocyte antigens, HLA) (Sullivan et al. 1997). Thus, additional genetic modifications, such as the transgenic expression of multiple HLA genes, will be necessary to inhibit NK-cell-mediated cytotoxicity in pig-to-human xenotransplantation.

In summary, we have shown that the co-injection of modified Cas9-D10A nickase with a pair of sgRNAs into the zygote’s cytoplasm can easily and efficiently generate biallelic modifications in pigs, in one step. Although the main disadvantage of the injection into the zygote is potential mosaicism of offspring, in our study, this did not occur.

ULBP1-KO pigs will provide a more progressive xenograft source for further research studies, especially those measuring the effects of abolishing the gene function in immunological interactions. At this moment, studies are being conducted double-track. The phenotype features of the first known ULBP1-KO pigs are being examined and crossbreeding of ULBP1-KO pigs with pigs expressing hHLA-E [data not published] is in progress. These two modifications together are probably necessary to protect porcine organs fully against human NK-mediated rejection after xenotransplantation.

## Abbreviations

NHPs: Non-human primates
HAR: Hyperacute rejection
DXR: Delayed xenograft rejection
hHT: Human α1,2-fucosyltransferase
GLA: Galactosyltransferase
hCRPs: Human complement regulatory proteins
NK: Natural killer
ADCC: Antibody-dependent cellular cytotoxicity
pULBP1: UL16-binding protein 1
sgRNA: Single-guide RNA
ZFNs: Zinc finger nucleases
TALENs: Transcription activator-like effector nucleases
CRISPR-Cas9: Clustered regularly interspaced short palindromic repeats
SCNT: Somatic cell nuclear transfer
GTKO: α1,3-Galactosyltransferase knockout
DSB: Double-strand breaks
HDR: Homologous directed repair
NHEJ: Non-homologous end-joining
